# Expression of a heat-stable NADPH-dependent alcohol dehydrogenase from *Thermoanaerobacter pseudethanolicus* 39E in *Clostridium thermocellum* 1313 results in increased hydroxymethylfurfural resistance

**DOI:** 10.1186/s13068-017-0750-z

**Published:** 2017-03-15

**Authors:** Sun-Ki Kim, Joseph Groom, Daehwan Chung, James Elkins, Janet Westpheling

**Affiliations:** 1 Department of Genetics, University of Georgia, Athens, GA USA; 2 Biosciences Center, National Renewable Energy Laboratory, Golden, CO USA; 3 Biosciences Division, Oak Ridge National Laboratory, Oak Ridge, TN USA; 4 BioEnergy Science Center, Oak Ridge National Laboratory, Oak Ridge, TN USA

**Keywords:** Consolidated bioprocessing, *Clostridium thermocellum*, Butanol dehydrogenase, Furfural, 5-hydroxymethyl-2-furfural

## Abstract

**Background:**

Resistance to deconstruction is a major limitation to the use of lignocellulosic biomass as a substrate for the production of fuels and chemicals. Consolidated bioprocessing (CBP), the use of microbes for the simultaneous hydrolysis of lignocellulose into soluble sugars and fermentation of the resulting sugars to products of interest, is a potential solution to this obstacle. The pretreatment of plant biomass, however, releases compounds that are inhibitory to the growth of microbes used for CBP.

**Results:**

Heterologous expression of the *Thermoanaerobacter pseudethanolicus* 39E *bdhA* gene, that encodes an alcohol dehydrogenase, in *Clostridium thermocellum* significantly increased resistance to furan derivatives at concentrations found in acid-pretreated biomass. The mechanism of detoxification of hydroxymethylfurfural was shown to be primarily reduction using NADPH as the cofactor. In addition, we report the construction of new expression vectors for homologous and heterologous expression in *C. thermocellum.* These vectors use regulatory signals from both *C. bescii* (the S-layer promoter) and *C. thermocellum* (the enolase promoter) shown to efficiently drive expression of the BdhA enzyme.

**Conclusions:**

Toxic compounds present in lignocellulose hydrolysates that inhibit cell growth and product formation are obstacles to the commercialization of fuels and chemicals from biomass. Expression of genes that reduce the effect of these inhibitors, such as furan derivatives, will serve to enable commercial processes using plant biomass for the production of fuels and chemicals.

**Electronic supplementary material:**

The online version of this article (doi:10.1186/s13068-017-0750-z) contains supplementary material, which is available to authorized users.

## Background

*Clostridium thermocellum* is a Gram-positive, thermophilic anaerobic bacterium and one of the most promising candidates for CBP because of its ability to deconstruct plant biomass and convert it directly to ethanol, lactic acid, acetic acid, formic acid, hydrogen, and amino acids including valine and alanine [1, 2]. While most metabolic engineering of *C. thermocellum* has focused on improving ethanol production [1, 3, 4], improving tolerance to inhibitors generated from biomass pretreatment is essential to make CBP by *C. thermocellum* an industrially relevant process [5]. Furfural, 2-furaldehyde, and HMF, 5-hydroxymethyl-2-furfural, are generated during pretreatment and inhibit both growth and fermentation by microorganisms [6], including *C. thermocellum*. *Saccharomyces cerevisiae* [7], *Escherichia coli* [8], and *Caldicellulosiruptor bescii* [9] can convert furfural and HMF to the less toxic alcohols, furfuryl alcohol and furan dimethanol, respectively. Overexpression of oxidoreductases, such as alcohol dehydrogenases (ADH1, ADH6, and ADH7) [7, 10, 11], a propanediol oxidoreductase (FucO) [8], and a butanol dehydrogenase (BdhA) [9] has been shown to increase specific furfural and HMF conversion rates. Among them, Teth39_1597 encoding the BdhA enzyme from *Thermoanaerobacter pseudethanolicus* 39E was shown to reduce both furfural and HMF at 60 °C using NADPH as the cofactor [12]. We recently demonstrated that heterologous expression of this heat-stable BdhA enzyme increased resistance of engineered *C. bescii* strains to both furfural and HMF [9]. *C. bescii* is a hyperthermophilic, Gram-positive, anaerobic bacterium that has the unusual ability to grow on a variety of lignocellulosic biomass substrates without conventional pretreatment [13, 14]. We recently engineered *C. bescii* to produce ethanol directly from switchgrass making it a strong candidate for CBP [15]. Pretreatment, however, increases rates of hydrolysis but releases furans that are toxic to growing cells. *C. thermocellum* relies primarily on pretreated biomass producing ethanol at high yield (72% of theoretical maximum) and produces ethanol as a single fermentation product [16, 17], making it perhaps the strongest candidate so far studied for CBP. To test whether BdhA from *T. pseudethanolicus* might also improve resistance to these compounds in *C. thermocellum*, we designed new expression vectors for *C. thermocellum*, using three different promoters, the *C. bescii* S-layer promoter, and the *C. thermocellum* Clo1313_1809 and enolase promoters. The vectors were based on the *C. bescii* replicon pBAS2 [18, 19]. Expression of BdhA in *C. thermocellum* resulted not only in increased resistance to HMF but also increased growth on cellulosic substrates and improved ethanol production. These data suggest that redox homeostasis in *C. thermocellum* plays an important role in its growth on cellulosic substrates.

## Results and discussion

### Heterologous expression of the *bdhA* gene from *T. pseudethanolicus* in *C. thermocellum*

**Fig. 1:**
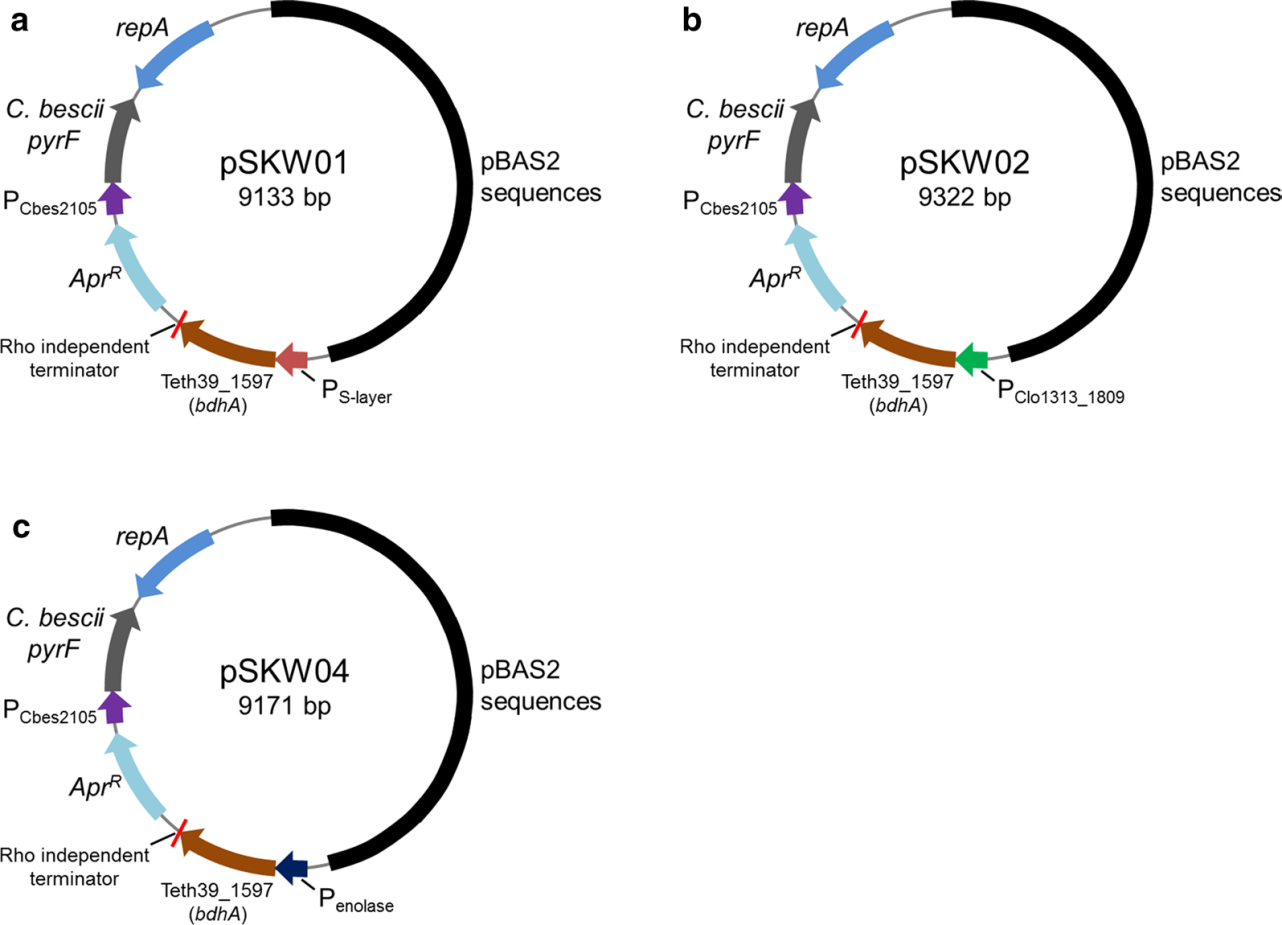
Maps of shuttle vectors for BdhA expression in *C. thermocellum*. The *bdhA* gene from *T. pseudethanolicus* 39E (Teth39_1597) was expressed under the control of the S-layer (**a**), Clo1313_1809 (**b**), and enolase (**c**) promoters. Shuttle vectors contain a C-terminal 6X His-tag version of *bdhA*, a Rho-independent terminator, the *pyrF* (from *C. bescii*) cassette for selection of plasmid transformants, and pBAS2 sequences for replication in *C. thermocellum*

Expression vectors for *bdhA* were based on plasmid pDCW89 [18] constructed from the native *C. bescii* plasmid pBAS2 [19] for use as an *E. coli/Caldicellulosiruptor* shuttle vector. This replicon is maintained stably in *C. thermocellum* at its optimal growth temperature of 60 °C [18]. Previous studies showed that the *C. bescii* S-layer [15, 20] and the *C. thermocellum* enolase [21] promoters were useful for expression of target genes in both *C. bescii* and *C. thermocellum*. For this study, the Clo1313_1809 promoter was also tested based on the fact that the steady state levels of RNA determined by transcriptional profiling (http://www.ncbi.nlm.nih.gov/geo/query/acc.cgi?acc=GSE54082) for genes under the control of this promoter were high. While steady state levels of RNA reflect both promoter strength and RNA stability, we selected this promoter as a possible candidate. The *bdhA* gene from *T. pseudethanolicus* 39E (Teth39_1597) was amplified by PCR and cloned under the transcriptional control of the *C. bescii* S-layer, Clo1313_1809, and *C. thermocellum* enolase (Cthe_0143) promoters. The P_S-layer_ -*bdhA* expression cassette containing a C-terminal 6X His-tag and a Rho-independent transcription terminator was cloned using plasmid pDCW89 as template to construct plasmid pSKW01 (Fig. 1a). pSKW02 and pSKW04 plasmids are identical to pSKW01 except for the promoter region, which contain Clo1313_1809 and *C. thermocellum* enolase promoters, respectively (Fig. 1b, c).

Plasmid DNA was transformed into a *pyrF* deletion mutant of *C. thermocellum* [22] and transformants were selected for uracil prototrophy. The presence of the plasmid in transformants was confirmed by PCR analysis (Additional file 1: Figure S1A). Primers (SK04 and DC228) were used to amplify the portion of the plasmid containing the open reading frame of *bdhA*, annealing to regions of the plasmid outside the *bdhA* gene. The expected PCR product was detected for pSKW01 and pSKW02 transformants but not for pSKW04 (Additional file 1: Figure S1A) suggesting that pSKW04 might have integrated into the *C. thermocellum* chromosome. To test whether the plasmids were replicating autonomously, total DNA isolated from *C. thermocellum* transformants containing pSKW01 (JWCT06), pSKW02 (JWCT07) or pSKW04 (JWCT08) was used to back-transform *E. coli*. Transformants were obtained for DNA from JWCT06 and JWCT07 but not JWCT08, again suggesting that while the plasmid was present it was not autonomously replicating. Two different digestions by restriction endonucleases performed on plasmid DNA purified from two independent *E. coli* back-transformant colonies resulted in identical digestion patterns relative to the original plasmids (Additional file 1: Figure S2), indicating that plasmids (pSKW01 and pSKW02) were autonomously replicating in *C. thermocellum* and were structurally stable during transformation and replication in *C. thermocellum* and back-transformation into *E. coli.* To test whether plasmid pSKW04 had integrated into the chromosome PCR amplification using various primers inside and outside plasmid sequences was used. The enolase promoter sequence is the only region of homology between the plasmid and the chromosome and the only potential site for homologous recombination. PCR amplification using a primer set specific for 0.1 kb upstream region of enolase promoter in the chromosome (SK038) and plasmid pSKW04 (DC461) (Additional file 1: Figure S1B) confirmed pSKW04 plasmid integration into the *C. thermocellum* chromosome at the site of the enolase promoter region via a single crossover event.

**Fig. 2:**
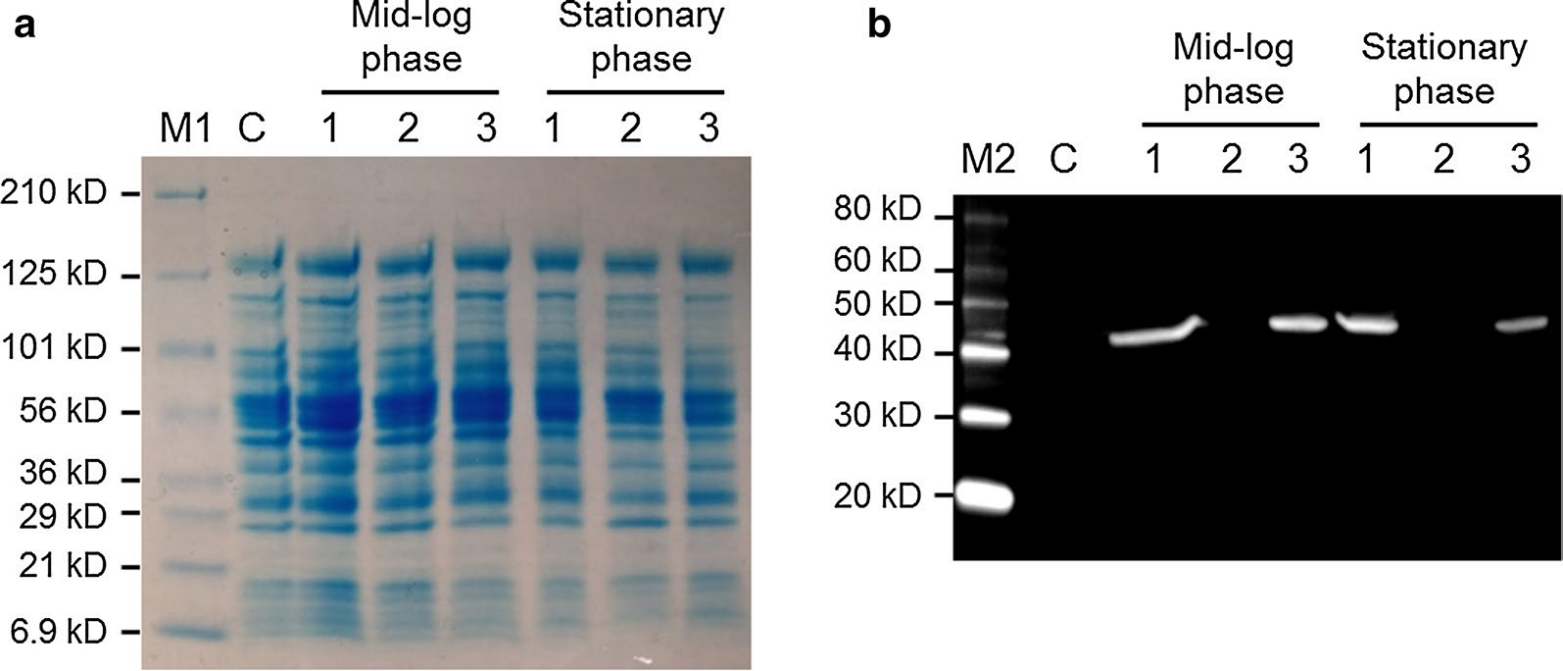
Confirmation of BdhA expression in *C. thermocellum*. Total cell lysates prepared from mid-log and stationary phases were electrophoresed either for SDS-PAGE analysis with Coomassie blue staining (**a**) or for Western blot analysis (**b**) probed with His-tag antibody. *C* JWCT02 (Δ*pyrF* + pDCW89); *1* JWCT06 (Δ*pyrF* + pSKW01); *2* JWCT07 (Δ*pyrF* + pSKW02); *3* JWCT08 (Δ*pyrF* + pSKW04); *M1* Pre-stained SDS-PAGE standards, Broad range (Bio-Rad Laboratories); *M2* MagicMark™ XP Western Protein Standard (Invitrogen)

To investigate expression of *T. pseudethanolicus* 39E BdhA in *C. thermocellum*, JWCT06, JWCT07, and JWCT08 strains were grown in CTFUD-NY medium without uracil. Although the BdhA protein (44 kD) was difficult to visualize using Coomassie blue staining (Fig. 2a), it was clearly visible by Western hybridization analysis using monoclonal anti-His antibodies (Fig. 2b). Of the three different expression systems, the constructs containing the *C. bescii* S-layer and *C. thermocellum* enolase promoters resulted in the best protein expression. We emphasize that detection of a protein product is an assay that combines transcription efficiency, RNA stability and protein stability and is not a direct assay of the promoters themselves. While expression levels of the P_S-layer_-*bdhA* cassette were similar throughout the mid-log and stationary phases, those of the P_enoalse_-*bdhA* cassette decreased slightly as cells entered stationary phase (Fig. 2b).

### Effects of BdhA expression on the growth and tolerance of *C. thermocellum* to furan derivatives

**Fig. 3:**
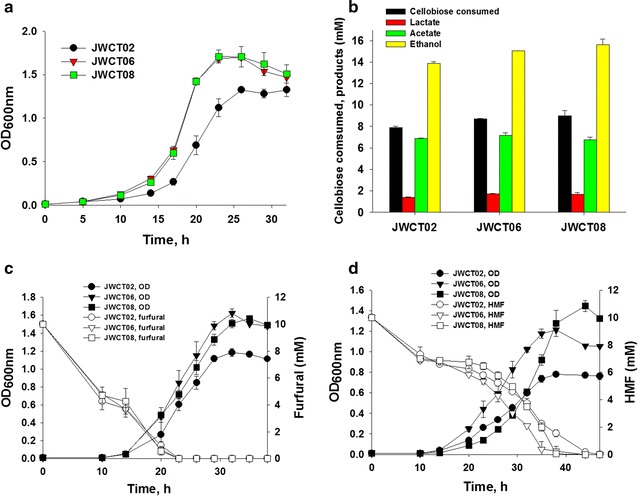
Batch fermentations of *C. thermocellum* strains in defined medium without or with fermentation inhibitors. **a** Cell growth of strains expressing BdhA compared to the control strain. **b** Fermentation products of *C. thermocellum* strains in defined medium without inhibitors. **c**, **d** Batch fermentations of *C. thermocellum* strains in defined medium containing 10 mM furfural (**c**) or HMF (**d**). JWCT02, the parent control strain; JWCT06, containing P_S-layer_ - *bdhA*; JWCT08, containing P_enolase_ -*bdhA*. Results are the mean of duplicate experiments and *error bars* indicate s.d

Interestingly, strains expressing the *bdhA* gene grew significantly better than the control strain. Maximum optical densities of strains expressing BdhA were 26% (JWCT06, *P*_*value*_ = 0.022) and 28% (JWCT08, *P*_*value*_ = 0.036) higher than the control strain in standard CTFUD-NY medium without furan aldehydes (Fig. 3a). In addition, volumetric ethanol production of JWCT06 and JWCT08 strains were 8% (*P*_*value*_ = 0.030) and 13% (*P*_*value*_ = 0.058) higher than the control strain with no effect on cellobiose consumption, lactate or acetate production (Fig. 3b). Previous studies reported that a complete loss of NADH-dependent activity by directed evolution of the AdhE enzyme, a bifunctional acetaldehyde-CoA/alcohol dehydrogenase, with concomitant acquisition of an NADPH-dependent activity conferred increased tolerance to ethanol in *C. thermocellum*, which likely affected the maintenance of NADP/NADPH pools linked to membrane changes [23]. The BdhA enzyme expressed in this study is also an NADPH-dependent alcohol dehydrogenase that does not use NADH as a cofactor [12]. This study and earlier studies suggest that redox homeostasis in *C. thermocellum* plays an important role in its growth and tolerance phenotypes.

**Fig. 4:**
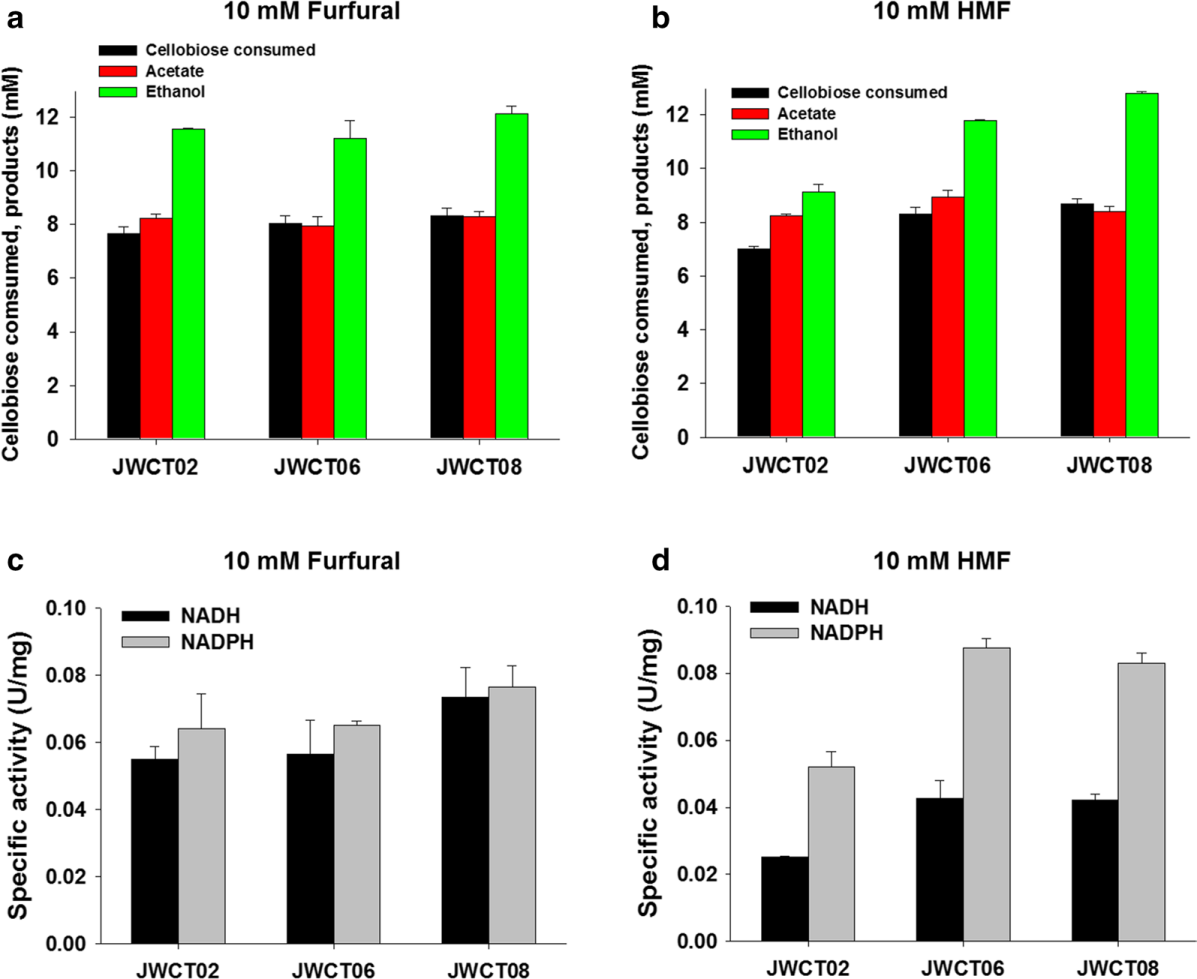
Comparison of fermentation products and in vitro reduction activity of furan derivatives by JWCT02, JWCT06, and JWCT08 strains. **a**, **b** Cellobiose consumed and fermentation products of *C. thermocellum*. JWCT02, JWCT06, and JWCT08 strains were grown in defined medium with 5 g/L cellobiose containing 10 mM furfural (**a**) or HMF (**b**). Results are the mean of duplicate experiments and *error bars* indicate s.d. **c**, **d** In vitro assays of reduction activity of furfural (**c**) or HMF (**d**). Crude protein extracts of JWCT02, JWCT06, and JWCT08 strains were assayed for reduction activity using NAD(P)H as cofactor. JWCT02, the parent control strain; JWCT06, containing P_S-layer_ -*bdhA*; JWCT08, containing P_enolase_ -*bdhA*. Results are the mean of triplicate experiments and *error bars* indicate s.d

To investigate the effects of BdhA expression on the tolerance of *C. thermocellum* to furan derivatives, we also performed fermentation experiments in the presence of 10 mM furfural or HMF. These compounds are present at approximately these concentrations in dilute acid pretreatment hydrolysates [5, 24]. As shown in Fig. 3c, conversion of furfural was rapid in all strains of *C. thermocellum,* but the strains expressing BdhA grew to significantly higher cell densities than the control strain. Maximum optical densities of strains expressing BdhA were 35% (JWCT06, *P*_*value*_ = 0.011) and 30% (JWCT08, *P*_*value*_ = 0.005) higher than the control strain in the presence of 10 mM furfural without affecting the amount of cellobiose consumed and end product concentrations (Fig. 4a). While both strains expressing BdhA were significantly more efficient at conversion of HMF (Fig. 3d), for strain JWCT08 growth was significantly better than either the control strain or strain JWCT06. Maximum optical densities of strains expressing BdhA were 54% (JWCT06, *P*_*value*_ = 0.0033) and 84% (JWCT08, *P*_*value*_ = 0.018) higher than the control strain in the presence of 10 mM HMF (Fig. 3d). Interestingly, conversion of HMF in strain JWCT06 was increased earlier and throughout growth phase compared to either the control strain or strain JWCT08. The JWCT06 strain consumed 18% (*P*_*value*_ = 0.030) more cellobiose and produced 29% (*P*_*value*_ = 0.025) more ethanol than the control JWCT02 strain with HMF present (Fig. 4b). The JWCT08 strain consumed 24% (*P*_*value*_ = 0.013) more cellobiose and produced 40% (*P*_*value*_ = 0.016) more ethanol than the control strain (Fig. 4b). These results show that BdhA expression increases resistance to HMF relative to the control strain. Addition of HMF decreased ethanol production and increased acetate production in the control strain compared to growth in the medium without inhibitors (Figs. 3b, 4b). Expression of BdhA led to reduced inhibition of ethanol production, and we speculate that acetate production might lead to an additional ATP per acetate, partially relieving ATP depletion caused by furan derivatives [25]. As shown in Fig. 4a, b, ethanol yield of the control JWCT02 strain in the presence of HMF was 21% (*P*_*value*_ = 0.027) lower than that in the presence of furfural. In contrast to previous studies showing that the toxic effect of furfural are greater than that of HMF in other microorganisms [6, 8, 9, 26], in this study, HMF was more inhibitory than furfural to the growth of *C. thermocellum*.

### In vitro NADH- and NADPH-dependent conversion activity of strains expressing BdhA

Aldehydes are toxic to microbial cell growth and cells convert these compounds to less toxic compounds such as alcohols and carboxylic acids. Previous studies reported that reduction activity was much higher than oxidation activity for detoxification of the furan derivatives [6, 8, 27]. To investigate the mechanism of conversion of furfural and HMF by strains expressing BdhA, we examined NADH- and NADPH-dependent activities of furfural and HMF reduction. Crude extracts from the BdhA expressing strains, JWCT06 and JWCT08, and the control strain (JWCT02) were prepared and in vitro reduction of furfural and HMF was measured. While specific activities of crude extracts of all strains toward furfural were similar, BdhA expression increased the reduction of HMF by 59–69% relative to the control (Fig. 4c, d). This result is likely due to the specificity of the BdhA enzyme as this enzyme is known to have a twofold higher activity on HMF than furfural [12]. We concluded from these data that the mechanism of improved detoxification rates of HMF by BdhA expression (Fig. 3d) results from higher reduction of HMF.

## Methods

### Bacterial strains, media, and culture conditions

*Clostridium thermocellum* and *E. coli* strains used in this study are listed in Table 1. All *C. thermocellum* strains were grown anaerobically in modified CTFUD medium [28], pH 7.0, with cellobiose (0.5% w/v) as the sole carbon source for routine growth and transformation experiments. *C. thermocellum* cells were grown at 60 °C, under an atmosphere of 85% nitrogen, 10% CO_2_, and 5% hydrogen. For uracil auxotrophs, 360 µM uracil was supplemented. *E. coli* BL21 (Invitrogen, Grand Island, NY, USA) grown in LB medium with 50 μg/mL apramycin was used for plasmid constructions.

**Table 1:** Strains and plasmids used in this study

Name	Description	Reference
*E. coli*		
JW513	BL21 containing pSKW01 (Apramycin^R^)	This study
JW514	BL21 containing pSKW02 (Apramycin^R^)	This study
JW515	BL21 containing pSKW04 (Apramycin^R^)	This study
*C. thermocellum*		
LL1005	*C. thermocellum* DSM 1313 Δ*pyrF* (*ura* ^−^/5-FOA^R^)	[25]
JWCT02	LL1005 containing pDCW89 (*ura* ^+^/5-FOA^S^)	[25]
JWCT06	LL1005 containing pSKW01 (*ura* ^+^/5-FOA^S^)	This study
JWCT07	LL1005 containing pSKW02 (*ura* ^+^/5-FOA^S^)	This study
JWCT08	LL1005 containing pSKW04 (*ura* ^+^/5-FOA^S^)	This study
Plasmids		
pDCW89	*E. coli*/*C. thermocellum* shuttle vector (Apramycin^R^)	[13, 25]
pDCW144	Intermediate vector 1 (Apramycin^R^)	[15]
pDCW148	Intermediate vector 2 (Apramycin^R^)	This study
pDCW171	Source of Teth39_1597 (*bdhA*) open reading frame (Apramycin^R^)	[9]
pSKW01	Expression vector containing P_S-layer_ -*bdhA* (Apramycin^R^)	This study
pSKW02	Expression vector containing P_Clo1313_1809_ -*bdhA* (Apramycin^R^)	This study
pSKW04	Expression vector containing P_enolase_ -*bdhA* (Apramycin^R^)	This study

### Construction and transformation of *bdhA* expression vectors

Plasmid DNA was isolated using a Qiagen Miniprep Kit (Qiagen, Valencia, CA, USA). Chromosomal DNA from *C. thermocellum* strains was extracted using the Quick-gDNA MiniPrep (Zymo Research, Irvine, CA, USA) according to the manufacturer’s instructions. Plasmids used in this study were constructed using Q5 High-Fidelity DNA polymerase (New England BioLabs, Ipswich, MA, USA) for PCR reactions, restriction enzymes (New England BioLabs, Ipswich, MA, USA), and the Fast-link DNA ligase kit (Epicentre Biotechnologies, Madison, WI, USA) according to the manufacturer’s instructions. Plasmid pSKW01 (Fig. 1a) was constructed in two cloning steps. First, the 2.8 kb Cthe0423 expression cassette containing the regulatory region of Cbes2303 (S-layer protein), a C-terminal 6X Histidine-tag, and a Rho-independent transcription terminator was amplified by PCR with primers DC460 (with an added PvuI site) and DC461 (with an added NotI site) using pDCW144 as template. A 7.7 kb DNA fragment containing the pSC101 replication origin for *E. coli*, a putative *C. thermocellum* replication origin, an apramycin resistance gene cassette (Apr^R^) and a *C. bescii pyrF* cassette was amplified with primers DC481 (with an added PvuI site) and DC482 (with an added NotI site) using pDCW89 as template. These two linear DNA fragments were digested with PvuI and NotI, and ligated to construct an 10.5 kb intermediate vector, pDCW148. In a second step, the 7.9 kb DNA fragment was amplified with primers DC576 (with and added PstI site) and DC466 (with an added SphI site, a 6X Histidine-tag, and a stop codon) using pDCW148 as a template. A 1.2 kb DNA fragment containing the coding sequence of *bdhA* (Teth39_1597) was amplified with DC577 (with an added PstI site) and DC578 (with an added SphI site) using pDCW171 as template. These two linear DNA fragments were digested with PstI and SphI, and ligated to construct a 9.1 kb plasmid, pSKW01. Plasmids pSKW02 and pSKW04 are identical to pSKW01 except for the promoter regions (Fig. 1). To make this change, a 0.3 kb DNA fragment containing the regulatory region of Clo1313_1809 was amplified with primers SK07 (with an added PstI site) and SK36 (with an added AvrII site) using *C. thermocellum* LL1005 genomic DNA (gDNA) as template. The 9.0 kb DNA fragment of pSKW01 without the regulatory region of Cbes2303 was amplified with primers SK04 (with an added PstI site) and SK28 (with an added AvrII site). These two linear DNA fragments were digested with PstI and AvrII, and ligated to construct a 9.3 kb plasmid, pSKW02 (Fig. 1b). In the case of plasmid pSKW04 (Fig. 1c), a 0.2 kb DNA fragment containing the enolase promoter region was amplified with primers SK19 (with an added PstI site) and SK26 (with an added AvrII site) using *C. thermocellum* LL1005 gDNA as template. *E. coli* BL21 cells were transformed by electroporation in a 1-mm-gap cuvette at 1.8 kV and transformants were selected for apramycin resistance. The sequences of all plasmids were verified by Automatic sequencing (Genewiz, South Plainfield, NJ, USA).

Electrotransformation of *C. thermocellum* cells was performed as previously described [29]. Cultures, electro-pulsed with plasmid DNA (~0.5 μg), were recovered in CTFUD+ C medium [29] at 60 °C. Recovery cultures were transferred to liquid CTFUD-NY medium [29] without uracil to allow selection of uracil prototrophs. Cultures were plated on solid CTFUD-NY media to obtain isolated colonies, and DNA was isolated from transformants. Taq polymerase (Sigma, St. Louis, MO, USA) was used for PCR reactions to confirm the presence of the plasmid. PCR amplification with primers (SK04 and DC228) outside the gene cassette on the plasmid was used to confirm the presence of the plasmid with the *bdhA* gene. In the case of the JWCT08 (*ΔpyrF* + pSKW04) strain, integration of plasmid pSKW04 after a single crossover in the enolase promoter region was verified by PCR amplification with primers SK038 (specific for 0.1 kb upstream region of enolase promoter in JWCT08 gDNA) and DC461 (specific for plasmid pSKW04). Primers used for plasmid constructions and confirmation are listed in Additional file 1: Table S1.

### Preparation of cell lysates and western blotting

*Clostridium thermocellum* strains (JWCT02, JWCT06, JWCT07, and JWCT08) were grown to mid-log or stationary phase at 60 °C in 20 mL CTFUD-NY medium without uracil. Cells were harvested by centrifugation at 6000×*g* at 4 °C for 15 min, and cell pellets were washed using 50 mM Tris–Cl buffer (pH 8.0) and resuspended in Tris–Cl buffer to OD_600_ 20. Cells were lysed by boiling in the presence of SDS [30]. Cell free extracts were electrophoresed in 4–15% gradient Mini-Protean TGX gels, that were either stained using Coomassie blue or were transferred to PVDF membranes (ImmobilonTM-P; EMD Millipore, Billerica, MA, USA) using a Bio-Rad Mini-Protean 3 electrophoretic apparatus and then probed with His-tag (6xHis) monoclonal antibody (1:5000 dilution; Invitrogen, Grand Island, NY, USA) using the ECL Western Blotting substrate Kit (Thermo Scientific, Waltham, MA, USA) as specified by the manufacturer.

### Fermentations

To test tolerance to various compounds, cultures of JWCT02, JWCT06, or JWCT08 strains were serially passaged every 24 h in 20 mL CTFUD-NY medium without uracil. After the second transfer, cultures were inoculated to the last culture to initial optical density (OD_600_) of 0.01. Batch fermentations were performed at 60 °C without agitation in 10 mL CTFUD-NY medium without uracil supplemented with either furfural or HMF at 0 or 10 mM concentrations. Optical cell density was monitored using a Jenway Genova spectrophotometer, measuring absorbance at 600 nm.

### Analytical methods

Cellobiose, glucose, acetate, lactate, ethanol, HMF, and furfural concentrations were determined by high-performance liquid chromatography (HPLC, Agilent Technologies 1200 Series). Metabolites were separated on an Aminex HPX-87H column (Bio-Rad Laboratories) at isocratic temperature (50 °C) and a flow (0.6 mL/min) rate in 5.0 mM H_2_SO_4_, and then passed through a refractive index detector (Agilent 1200 Infinity Refractive Index Detector). Peak areas and retention times were compared to known standards of the same analyte.

### Enzyme activity assays

To prepare protein extracts, *C. thermocellum* cells were grown at 60 °C in 20 mL CTFUD-NY medium without uracil to an OD_600_ of 0.7–0.9, harvested by centrifugation at 6000×*g* at 4 °C for 10 min, suspended in CelLytic B cell lysis reagent (Sigma, USA), and lysed by a combination of 4X freeze-thawing and sonication (3 times for 15 s at 40 amps with 1 min rests on ice). Samples were centrifuged to separate protein lysate from cell debris, and the supernatants were used as protein extracts. To determine reduction activity of HMF and furfural, the reaction solution was formulated with 500 μL of 100 mM potassium phosphate buffer (pH 7.2), 300 μL of 33 mM furfural or HMF, and 100 μL of the crude enzyme solution. The absorbance change at 60 °C and 340 nm wavelength was monitored by a Jenway Genova spectrophotometer after addition of 100 μL of 1 mM NAD(P)H. One unit of reduction activity was defined as the amount of enzyme oxidizing 1 μmol NAD(P)H per minute. Protein concentrations were determined using the Bio-Rad protein assay kit.

## Additional file

**Additional file: Table S1.** List of primers used in this study. **Figure S1.** Confirmation of expression vector transformation in *C. thermocellum*. **Figure S2.** Verification of the stable presence of shuttle vectors in *C. thermocellum* transformants.

## Abbreviations

5-FOA: 5-fluoroorotic acid
LB broth: Luria Bertani broth
SDS: sodium lauryl sulfate
OD: optical density
HMF: 5-hydroxymethyl-2-furfural
CBP: consolidated bioprocessing
HPLC: high-performance liquid chromatography

## References

[1] Deng Y, Olson DG, Zhou JL, Herring CD, Shaw AJ, Lynd LR (2013). Redirecting carbon flux through exogenous pyruvate kinase to achieve high ethanol yields in *Clostridium thermocellum*. Metab Eng.

[2] Akinosho H, Yee K, Close D, Ragauskas A (2014). The emergence of *Clostridium thermocellum* as a high utility candidate for consolidated bioprocessing applications. Front Chem..

[3] Papanek B, Biswas R, Rydzak T, Guss AM (2015). Elimination of metabolic pathways to all traditional fermentation products increases ethanol yields in *Clostridium thermocellum*. Metab Eng.

[4] Biswas R, Zheng TY, Olson DG, Lynd LR, Guss AM (2015). Elimination of hydrogenase active site assembly blocks H_2_ production and increases ethanol yield in *Clostridium thermocellum*. Biotechnol Biofuels.

[5] Almeida JRM, Modig T, Petersson A, Hahn-Hagerdal B, Liden G, Gorwa-Grauslund MF (2007). Increased tolerance and conversion of inhibitors in lignocellulosic hydrolysates by *Saccharomyces cerevisiae*. J Chem Technol Biotechnol.

[6] Kim SK, Jin YS, Choi IG, Park YC, Seo JH (2015). Enhanced tolerance of *Saccharomyces cerevisiae* to multiple lignocellulose-derived inhibitors through modulation of spermidine contents. Metab Eng.

[7] Liu ZL, Moon J, Andersh BJ, Slininger PJ, Weber S (2008). Multiple gene-mediated NAD(P)H-dependent aldehyde reduction is a mechanism of in situ detoxification of furfural and 5-hydroxymethylfurfural by *Saccharomyces cerevisiae*. Appl Microbiol Biotechnol.

[8] Wang X, Yomano LP, Lee JY, York SW, Zheng HB, Mullinnix MT, Shanmugam KT, Ingram LO (2013). Engineering furfural tolerance in *Escherichia coli* improves the fermentation of lignocellulosic sugars into renewable chemicals. Proc Natl Acad Sci USA.

[9] Chung D, Verbeke TJ, Cross KL, Westpheling J, Elkins JG (2015). Expression of a heat-stable NADPH-dependent alcohol dehydrogenase in *Caldicellulosiruptor bescii* results in furan aldehyde detoxification. Biotechnol Biofuels.

[10] Almeida JRM, Roder A, Modig T, Laadan B, Liden G, Gorwa-Grauslund MF (2008). NADH- vs NADPH-coupled reduction of 5-hydroxymethyl furfural (HMF) and its implications on product distribution in *Saccharomyces cerevisiae*. Appl Microbiol Biotechnol.

[11] Petersson A, Almeida JRM, Modig T, Karhumaa K, Hahn-Hagerdal B, Gorwa-Grauslund MF, Liden G (2006). A 5-hydroxymethyl furfural reducing enzyme encoded by the *Saccharomyces cerevisiae ADH6* gene conveys HMF tolerance. Yeast.

[12] Clarkson SM, Hamilton-Brehm SD, Giannone RJ, Engle NL, Tschaplinski TJ, Hettich RL, Elkins JG (2014). A comparative multidimensional LC-MS proteomic analysis reveals mechanisms for furan aldehyde detoxification in *Thermoanaerobacter pseudethanolicus* 39E. Biotechnol Biofuels.

[13] Dam P, Kataeva I, Yang SJ, Zhou FF, Yin YB, Chou WC, Poole FL, Westpheling J, Hettich R, Giannone R (2011). Insights into plant biomass conversion from the genome of the anaerobic thermophilic bacterium *Caldicellulosiruptor bescii* DSM 6725. Nucleic Acids Res.

[14] Yang SJ, Kataeva I, Hamilton-Brehm SD, Engle NL, Tschaplinski TJ, Doeppke C, Davis M, Westpheling J, Adams MW (2009). Efficient degradation of lignocellulosic plant biomass, without pretreatment, by the thermophilic anaerobe “*Anaerocellum thermophilum*” DSM 6725. Appl Environ Microbiol.

[15] Chung D, Cha M, Guss AM, Westpheling J (2014). Direct conversion of plant biomass to ethanol by engineered *Caldicellulosiruptor bescii*. Proc Natl Acad Sci USA.

[16] Olson DG, McBride JE, Shaw AJ, Lynd LR (2012). Recent progress in consolidated bioprocessing. Curr Opin Biotechnol.

[17] Lo J, Olson DG, Murphy SJ, Tian L, Hon S, Lanahan A, Guss AM, Lynd LR (2017). Engineering electron metabolism to increase ethanol production in *Clostridium thermocellum*. Metab Eng.

[18] Chung D, Cha M, Farkas J, Westpheling J (2013). Construction of a stable replicating shuttle vector for *Caldicellulosiruptor* species: use for extending genetic methodologies to other members of this genus. PLoS ONE.

[19] Clausen A, Mikkelsen MJ, Schroder I, Ahring BK (2004). Cloning, sequencing, and sequence analysis of two novel plasmids from the thermophilic anaerobic bacterium *Anaerocellum thermophilum*. Plasmid.

[20] Chung D, Young J, Bomble YJ, Vander Wall TA, Groom J, Himmel ME, Westpheling J (2015). Homologous expression of the *Caldicellulosiruptor bescii* CelA reveals that the extracellular protein is glycosylated. PLoS ONE.

[21] Olson DG, Maloney M, Lanahan AA, Hon S, Hauser LJ, Lynd LR (2015). Identifying promoters for gene expression in *Clostridium thermocellum*. Metab Eng Commun..

[22] Tripathi SA, Olson DG, Argyros DA, Miller BB, Barrett TF, Murphy DM, McCool JD, Warner AK, Rajgarhia VB, Lynd LR (2010). Development of *pyrF*-based genetic system for targeted gene deletion in *Clostridium thermocellum* and creation of a *pta* mutant. Appl Environ Microbiol.

[23] Brown SD, Guss AM, Karpinets TV, Parks JM, Smolin N, Yang SH, Land ML, Klingeman DM, Bhandiwad A, Rodriguez M (2011). Mutant alcohol dehydrogenase leads to improved ethanol tolerance in *Clostridium thermocellum*. Proc Natl Acad Sci USA.

[24] Hsu TC, Guo GL, Chen WH, Hwang WS (2010). Effect of dilute acid pretreatment of rice straw on structural properties and enzymatic hydrolysis. Bioresour Technol.

[25] Ask M, Bettiga M, Mapelli V, Olsson L (2013). The influence of HMF and furfural on redox-balance and energy-state of xylose-utilizing *Saccharomyces cerevisiae*. Biotechnol Biofuels.

[26] Franden MA, Pienkos PT, Zhang M (2009). Development of a high-throughput method to evaluate the impact of inhibitory compounds from lignocellulosic hydrolysates on the growth of *Zymomonas mobilis*. J Biotechnol.

[27] Park SE, Koo HM, Park YK, Park SM, Park JC, Lee OK, Park YC, Seo JH (2011). Expression of aldehyde dehydrogenase 6 reduces inhibitory effect of furan derivatives on cell growth and ethanol production in *Saccharomyces cerevisiae*. Bioresour Technol.

[28] Olson DG, Lynd LR (2012). Chap. 17: transformation of *Clostridium thermocellum* by electroporation. Methods Enzymol.

[29] Groom J, Chung D, Olson DG, Lynd LR, Guss AM, Westpheling J (2016). Promiscuous plasmid replication in thermophiles: use of a novel hyperthermophilic replicon for genetic manipulation of *Clostridium thermocellum* at its optimum growth temperature. Metab Eng Comm..

[30] Tsugama D, Liu SK, Takano T (2011). A rapid chemical method for lysing *Arabidopsis* cells for protein analysis. Plant Methods..

