# Supplementary material for: Expression of a heat-stable NADPH-dependent alcohol dehydrogenase from Thermoanaerobacter pseudethanolicus 39E in Clostridium thermocellum 1313 results in increased hydroxymethylfurfural resistance
Source: Biotechnol Biofuels. 2017 Mar 15;10:66. doi: 10.1186/s13068-017-0750-z (PMC5353787; doi:10.1186/s13068-017-0750-z)
Supplement: Supplementary file 1 — Additional file: Table S1. List of primers used in this study. Figure S1. Confirmation of expression vector transformation in C. thermocellum. Figure S2. Verification of the stable presence of shuttle vectors in C. thermocellum transformants. [file 13068_2017_750_MOESM1_ESM.docx]

Table S1. List of primers used in this study. The italicized sequences indicate the recognition sites of the corresponding restriction enzymes.

| Name | Sequence (5’ → 3’) | Restriction enzyme | Description |
| --- | --- | --- | --- |
| DC460 | AGAGAG*CGATCG*ACAGTTTGATTACAGTTTAGTCAGAGCT | PvuI | To construct pDCW148 |
| DC461 | AGAAGAAG*GCGGCCGC*TTGGTTCCTTAAATCTAAGAGGTATGA | NotI |  |
| DC481 | AGACTC*CGATCG*ATTCCCATGAGCCCACGAACAGT | PvuI | To construct pDCW148 |
| DC482 | AGAAGAAG*GCGGCCGC*TCTGACGCTCAGTGGAACGAA | NotI |  |
| DC576 | ACTACT*CTGCAG*CTCACCAAACCTCCTTGTATGAT | PstI | To construct pSKW01 |
| DC466 | AGA*GCATGC*CATCACCATCACCATCACTAATAATAAAGCTGAAATAAAAGAGGG | SphI |  |
| DC577 | ACTACT*CTGCAG* ATGATGAAATTTGAATTTTATAACCCGACC | PstI | To construct pSKW01 |
| DC578 | AGA*GCATGC*CATTGCCATGCGTAGTATTTCTA | SphI |  |
| SK07 | ACT*CTGCAG* ACCTCCCGTTAATTAATTTAAAGCATTAGAAAATGAAGC | PstI | To construct pSKW02 |
| SK36 | AGA*CCTAGG*ACTCCTCATTCAGTACTACTTATTCG | AvrII |  |
| SK04 | ACT*CTGCAG*ATGATGAAATTTGAATTTTATAACCCGACCCGAC | PstI | To construct pSKW02 & 04 |
| SK28 | AGA*CCTAGG*ATTCCCATGAGCCCACGAACAGT | AvrII |  |
| SK19 | ACT*CTGCAG*TTCTCCCTTCATATAGCTTATTTTTATATGTTAAAAAAT | PstI | To construct pSKW04 |
| SK26 | AGA*CCTAGG*GGAAATATTAAAATGGAAATGTTGAAAAAATGTTTTAAG | AvrII |  |
| SK04 | ACT*CTGCAG*ATGATGAAATTTGAATTTTATAACCCGACCCGAC | PstI | To confirm transformants |
| DC228 | ATCATCCCCTTTTGCTGATG | - |  |
| SK038 | TCTCAGCACTTTTGATAATTCTGACTGTG | - | To confirm integration |
| DC461 | AGAAGAAG*GCGGCCGC*TTGGTTCCTTAAATCTAAGAGGTATGA | NotI |  |

**
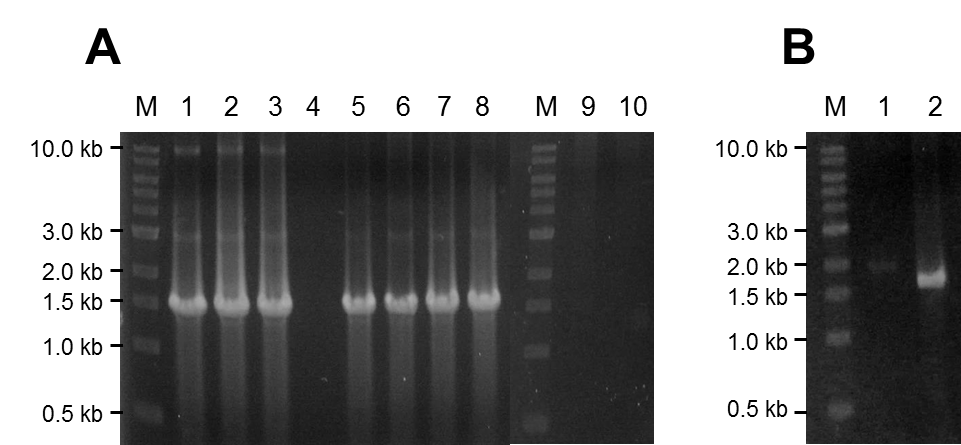
**

**Figure S1. Confirmation of expression vector transformation in *C. thermocellum*.** (A) Gel showing the 1.4 kb PCR products using plasmid-specific primers SK04 and DC228. 1: plasmid pSKW01; 2: plasmid pSKW02; 3: plasmid pSKW04; 4: negative control LL1005 (*△pyrF*); 5 and 6: JWCT06 (*△pyrF +* pSKW01); 7 and 8: JWCT07 (*△pyrF +* pSKW02); 9 and 10: JWCT08 (*△pyrF +* pSKW04). (B) Gel showing the 1.6 kb PCR product amplified from enolase promoter region in the chromosome using integration-specific primers SK038 and DC461. 1: negative control JWCT02 (*△pyrF +* pDCW89); 2: JWCT08; M: NEB 1 kb DNA ladder.


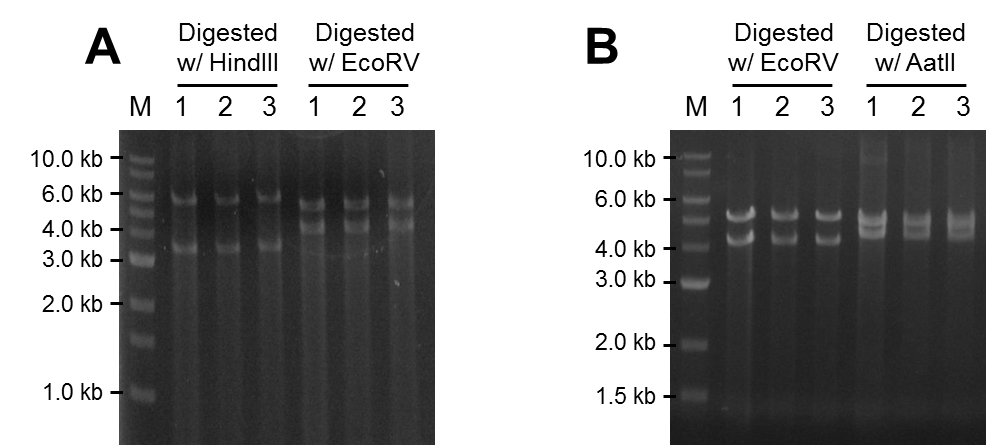


**Figure S2. Verification of the stable presence of shuttle vectors in *C. thermocellum* transformants.** (A) Restriction analysis of pSKW01 plasmid DNA before and after transformation of *C. thermocellum* and back-transformation to *E. coli*. 1: pSKW01 plasmid DNA isolated from *E. coli* BL21, digested with either HindIII (5.8 kb and 3.4 kb cleavage products), or with EcoRV (5.1 kb and 4.0 kb cleavage products), 2 and 3 : plasmid DNA isolated from two biologically independent *E. coli* BL21 back-transformants using total DNA isolated from *C. thermocellum* transformants, digested with either HindIII or EcoRV. (B) Restriction analysis of pSKW02 plasmid DNA before and after transformation of *C. thermocellum* and back-transformation to *E. coli*. 1: pSKW02 plasmid DNA isolated from *E. coli* BL21, digested with either EcoRV (5.1 kb and 4.2 kb cleavage products), or with AatII (4.9 kb and 4.4 kb cleavage products); 2 and 3: plasmid DNA isolated from two biologically independent *E. coli* BL21 back-transformants using total DNA isolated from *C. thermocellum* transformants, digested with either EcoRV or AatII; M: NEB 1 kb DNA ladder.
